# *Mesorhizobium salmacidum* sp. nov. and *Mesorhizobium argentiipisi* sp. nov. are symbionts of the dry-land forage legumes *Lessertia diffusa* and *Calobota sericea*

**DOI:** 10.1007/s10482-025-02063-2

**Published:** 2025-02-12

**Authors:** Esther K. Muema, Melandré van Lill, Stephanus N. Venter, Wai Yin Chan, Ricu Claassens, Emma T. Steenkamp

**Affiliations:** 1 Department of Biochemistry, Genetics and Microbiology, Forestry and Agricultural Biotechnology Institute (FABI), University of Pretoria, Pretoria, 0002 South Africa; 2 Department of Soil Science, Faculty of AgriSciences, Stellenbosch University, Matieland, South Africa

**Keywords:** Multilocus sequence analysis, Nodulation, Rhizosphere soils, Symbiotic loci, Up-to-date bacteria core gene

## Abstract

Legumes *Lessertia diffusa* and *Calobota sericea*, indigenous to South Africa, are commonly used as fodder crops with potential for sustainable livestock pasture production. Rhizobia were isolated from their root nodules grown in their respective soils from the Succulent Karoo biome (SKB) in South Africa, identified and characterized using a polyphasic approach. Sequence analysis of the 16S rRNA gene confirmed all isolates as *Mesorhizobium* members, which were categorized into two distinct lineages using five housekeeping protein-coding genes. Lineage I included 14 strains from both legumes, while Lineage II comprised a single isolate from *C. sericea*. Differences in phenotypic traits were observed between the lineages and corroborated by average nucleotide identity analyses. While all strains nodulated their original hosts, strains from *C. sericea* failed to effectively nodulate *L. diffusa* and vice versa. Phylogenetic analyses of nitrogen fixation (*nifH*) and nodulation (*nodA*, *nodC*) loci grouped all strains in a single clade, suggesting that unique symbiotic loci determine nodulation of these legumes. We designated Lineage I and II as *Mesorhizobium salmacidum* sp. nov. (Ld1326^Ts^; GCA_037179605.1^Ts^) and *Mesorhizobium argentiipisi* sp. nov. (Cs1330R2N1^Ts^; GCA_037179585.1^Ts^), using genome sequences as nomenclatural types according to the Nomenclatural Code for Prokaryotes using Sequence Data, thus avoiding complications with South Africa's biodiversity regulations. Identifying effective microsymbionts of *L. diffusa* and *C. sericea* is essential for conservation of Succulent Karoo Biome, where indigenous invasive species like *Vachellia karroo* and non-native Australian acacia species are present. Furthermore, targeted management practices using effective symbionts of the studied legumes can sustain the biome's socio-economic contribution through fodder provision.

**Supplementary Information:**

The online version contains supplementary material available at 10.1007/s10482-025-02063-2.

## Introduction

The papilionoid legumes *Calobota sericea* and *Lessertia diffusa* are included among the species prioritized for use as forage crops in water-limited agricultural systems, especially in southern Africa (Müller et al. 2017, Trytsman 2013). Both species are adapted to drought or arid conditions, as well as to rocky or sandy soils with pH ranging from 4.9 to 8.4 (Müller et al. 2017, Trytsman 2013, Muema et al. 2022). Accordingly, these legumes are regarded as particularly important forage crops for livestock production in the Western and Northern Cape Provinces of South Africa (Müller et al. 2017). For example, the arid to semi-arid Succulent and Nama Karoo regions range from being deserts to xeric shrublands, where the available soils are typically underdeveloped or sandy, lime-rich and nutrient-poor, with low water-holding capacity (Low and Rebelo 1998).

When selecting and developing legumes as forage crops in water-stressed and nutrient-poor soils, biological or symbiotic nitrogen fixation is an important consideration, given legumes’ ability to associate with bacterial symbionts capable of nitrogen fixation (Grahma and Vance 2003; Sprent and Gehlot 2010). These specialized bacteria are commonly referred to as “rhizobia” and induce on their hosts the formation of root- and/or stem-nodules in that they catalyze the conversion of atmospheric dinitrogen to plant-usable ammonium, a service they provide in return for energy-rich photosynthates (Poole and Allaway 2000). Cultivated legumes are thus often treated with compatible rhizobial inoculants to ensure the benefits of biological nitrogen fixation and to avoid the need for commercial nitrogen fertilizers (Herridge 2008). Although rhizobial inoculants are not available for *C. sericea* and *L. diffusa*, previous work showed that various strains of *Mesorhizobium*, *Ensifer*/*Sinorhizobium* and *Bradyrhizobium* are symbionts of *C. sericea*, while those of *L. diffusa* include strains of *Mesorhizobium* and *Rhizobium* (Gerding et al. 2012, Gerding et al. 2013, Gerding et al. 2014; Phalane et al. 2008). However, none of these rhizobial strains have been identified to species level.

Accurate and robust procedures for identifying or recognizing specific rhizobia are integral to the selection and analysis of strains that could potentially serve as inoculants (Mendoza-Suárez et al. 2021, Herridge 2008, Yates et al. 2011). Traditionally, this was achieved using complex and labor-intensive strain identification methods (Mendoza-Suárez et al. 2021; Batista et al. 2015), but the increasing availability of genome sequences makes it possible to design rapid and simple methods for recognizing specific rhizobial strains and their symbiotic loci (Ferguson et al. 2020; Yates et al. 2011). Indeed, as an essential requirement, all descriptions of rhizobial species must now be accompanied by publicly available whole genome sequences for at least the type strains (De Lajudie et al. 2019). Therefore, such genomic resources can be used to develope high-resolution marker systems to track rhizobial strains along the entire inoculant production workflow, from initial isolation and testing of elite strains under field conditions, through to inoculant production and quality control (Mendoza-Suárez et al. 2021, Herridge 2008).

Despite an increase in rhizobial diversity studies and the increased availability of genome sequence information during the last decade, the symbionts of legumes not cultivated at commercial scales are vastly understudied, and even fewer have been characterized and named (León-Barrios et al. 2021; Janczarek et al. 2024; Wulandari et al. 2024). Also, in countries such as South Africa and India where strict and complex legislation have been adopted to regulate biodiversity resources (Hamer et al. 2021; Alexander et al. 2021), formal description of rhizobial species is hindered as researchers from these countries typically cannot comply with the rules of the regulatory body governing bacterial nomenclature (Da Silva et al. 2023; Garrity et al. 2015, Rahi 2021). A potential solution to this hinderance is to describe strains from these countries following the recently published Code of Nomenclature of Prokaryotes Described from Sequence Data (SeqCode) (Hedlund et al. 2022; Whitman et al. 2024). Validation of a type sequence (denoted by “Ts”) under the SeqCode allows a genome sequence to serve as the nomenclatural type, thus eliminating the need to deposit axenic cultures in at least two international culture collections (van Lill et al. 2024; Hedlund et al. 2022).

In the current study, our goal was to isolate, characterize and provide descriptions for rhizobial symbionts of *C. sericea* and *L. diffusa* for validation using the SeqCode. For this purpose, we specifically targeted soils in the root zone in which rhizobia were known to represent substantial proportions of the resident microbial communities (Muema et al. 2022). Bacterial strains obtained from nodules on plant roots grown in these soils were then analysed using a polyphasic approach that incorporates genealogical concordance analysis for identifying putative species, and corroboration of their plausibility using genomic and phenotypic information (Venter et al. 2017). To explore the origins of their symbiotic loci, phylogenetic analyses of nitrogen fixation gene *nifH* and nodulation genes *nodA* and *nodC* were conducted. The dinitrogenase reductase component of the nitrogenase enzyme complex is encoded by *nifH,* while *nodA* and *nodC*, respectively, encode N-acyltransferase and N-acetylglucosaminyltransferase determining synthesis of nodulation factor (NF) (Bevan 2006). Finally, we aimed to provide descriptions for the novel rhizobial species following the principles outlined in the SeqCode, using genome sequences as nomenclatural types (Whitman et al. 2022, 2024; Hedlund et al. 2022). The work presented here would therefore demonstrate how this new nomenclatural code may be applied for describing rhizobial species of agricultural importance, as well as highlight the need for employing genome-informed taxonomic frameworks for recognizing such species and strains.

## Materials and methods

### Sample collection, soil properties and rhizobial isolation

Four composite soil samples used for trapping the rhizobia examined in this study were collected during a previous study (Muema et al. 2022) from two locations in the Northern Cape Province South Africa. Soil samples Cs1299 and Cs1321 (*C. sericea* rhizosphere) and Ld1326 (*L. diffusa* rhizosphere) originated from Brakputs (29°54′19.43" S, 17°34′37.41" E), while sample Cs1330 (*C. sericea* rhizosphere) came from Kamieskroon (30°17′15.74" S, 17°51′43.81" E). Both legume species are indigenous to the region (Müller et al. 2017) and soils, together with seeds for the two species, were collected with land owner permission.

Each composite soil sample was split into two parts, one of which was sieved (2 mm mesh) and used for soil analysis at Bemlab (Cape Town, South Africa). The parameters determined included pH with the KCl method, total carbon (TC) and nitrogen (TN) using the dry combustion method. Bemlab also determined the levels of ammonium (NH_4_^+^) and nitrates (NO_3_^−^) calorimetrically, as well as soil texture using the Hydrometer method.

The second part of each composite sample was used for growing the respective legumes from which root nodules were obtained. These experiments were conducted under controlled conditions. To allow germination in the laboratory, *C. sericea* seeds were pretreated with concentrated sulphuric acid for 15–30 min, followed by six washes with sterile water. For *L. diffusa*, seeds were scarified with sandpaper, surface disinfested for 30 min with 80% (v/v) ethanol, and rinsed with sterile distilled water (Howieson and Dilworth 2016; Gerding et al. 2014). All pretreated seeds were imbibed in sterile water for 3–4 h and placed onto Petri dishes containing water agar (Merck; 15 g L^−1^) and incubated in the dark at 15 °C for 5 to 6 days.

After germination of *C. sericea* seeds, Leonard jars containing sterile nitrogen free Hoagland’s solution and sand (Somasegaran and Hoben 1994; Howieson and Dilworth 2016) were prepared by scooping out approximately 20 g of sand and replacing it with an equal volume of a particular rhizosphere soil sample. Three seedlings of *C. sericea* were planted into each of the respective Leonard jars prepared for the three samples (Cs1299, Cs1321 and Cs1330) and covered with glass Petri dish lids. In the case of *L. diffusa*, approximately 25 g of the Ld1326 rhizosphere soil was placed on a layer of sterilised sand in a 50 ml Falcon tube within which we then planted one seedling and replaced the tube’s lid loosely. Once the plants had reached appreciable heights, the glass Petri dish or Falcon tubes lids were removed, and sterile sand coated with paraffin wax spread on top of the jar/tube contents around the pant stem to reduce moisture loss. Control jars/tubes (without rhizosphere soil) for each legume species were included. The glass house was set at 28 °C for 14 h daytime and 15 °C for 10 h nighttime. Plants were grown for 6–8 weeks after which nodules were harvested. This experiment was replicated at least twice for *C. sericea* soil samples and thrice for the *L. diffusa* soil sample.

Roots were carefully washed to remove any sand/soil, and the nodules excised. For isolation of the rhizobia, nodules were surface sterilized for 2–3 min using 3.5% (v/v) sodium hypochlorite and rinsed 5 times in sterile water. Individual nodules were then squeezed using sterile forceps and the extract streaked with a sterile inoculation loop onto yeast mannitol agar (YMA; Merck) containing Congo red (diphenyldiazo-bis-α-naphthylamine sulfonate) (Somasegaran and Hoben 1994), and incubated at 28 °C for approximately 5–7 days. Isolates were streaked onto fresh YMA and/or tryptone yeast agar (TYA; Merck) and incubated at 28 °C for 5–7 days, after which single colonies were used to establish pure cultures. The latter were stored at −70 °C using sterile 20% (v/v) glycerol as a cryoprotectant.

### Delineation of presumptive rhizobial species

For identifying the root-nodule bacteria isolated in this study, various DNA-based analyses were performed. This was achieved by subjecting YMA or TYA cultures of each isolate to DNA extraction with the Quick-gDNA mini prep kit (Zymo Research, Inqaba Biotechnical Industries Proprietary Limited, Pretoria, South Africa). The DNA extracts were then used as templates for PCR amplification of portions of the 16S ribosomal RNA (rRNA) gene and five housekeeping genes (see Table 1 for primers details and PCR conditions). The housekeeping genes were *atpD* (encoding ATP synthase β-subunit), *recA* (encoding homologous recombination protein A), *dnaK* (encoding chaperone protein DnaK), *glnII* (encoding Glutamine Synthetase II) and *rpoB* (encoding DNA polymerase β-subunit).

**Table 1:** Details of primers and PCR cycling conditions used in this study

Primers	Sequences	PCR conditions	References
16S rRNA 27F 16S rRNA 1492R	5'-AGAGTTTGATCCTGGCTCAG-3' 5'-CTACGGCTACCTTGTTACG-3'	94 °C for 2 min, (94 °C for 1 min 55 °C for 1 min,72 °C for 1 min) × 30, 72 °C for 7 min	(Heuer et al. 1997)
*atpD* 255F *atpD* 782R	5'-GCTSGGCCGCATCMTSAACGTC-3' 5'-GCC GAC ACT TCM GAA CCN GCC TG-3'	94 °C for 5 min, (95 °C for 45 secs, 59 °C for 30 s, 72 °C for 1 min 50 secs) × 30, 72 °C for 7 min	(Vinuesa et al. 2005)
*recA*-41F *recA*-640R	5'-TTC GGC AAG GGM TCG RTS ATG-3' 5'-ACA TSA CRC CGA TCT TCA TCG-3'	95 °C for 5 min, (94 °C for 30 secs, 62 °C for 15 secs, 72 °C for 45 secs) × 35, 72 °C for 5 min	(Vinuesa et al. 2005)
TS*dnaK*3 TS*dnaK*2	5'-AAGGAGCAGCAGATCCGCATCCA-3' 5'-GTACATGGCCTCGCCGAGCTTCA-3'	94 °C for 4 min, (94 °C for 1 min, 62 °C for 1 min, 72 °C for 40 secs) × 35, 72 °C for 5 min	(Stępkowski et al. 2003)
*glnII* 12F *glnII* 689R	5'-YAAGCTCGAGTACATYTGGCT-3' 5'-TGCATGCCSGAGCCGTTCCA-3'	94 °C for 5 min, (94 °C for 30 secs, 58 for 45 secs, 72 for 1 min) × 35, 72 °C for 10 min	(Vinuesa et al. 2005)
*rpoB*82F *rpoB*1580R	5'-AAC CTC ATC GAG GTT CAG AAG GC-3' 5'-TGG TCC ATG AAC TGC GAG AGC TG-3'	95 °C for 5 min, (94 °C for 2 min, 58 for 2 min, 72 °C for 1 min) × 3, (94 °C for 30 secs, 58 °C for 1 min, 72 °C for 1 min) × 30, 72 °C for 5 min	(Capela et al. 2001)

Each 25 µl PCR mixture contained 20 ng μl^−1^ template DNA, 0.2 mM of each dNTP, 0.2 μM of each primer, 2.0 mM MgCl_2_, 10 mg ml^−1^ bovine serum albumin (Merck), and 2 U Super-Therm *Taq* polymerase and Super-Therm *Taq* polymerase reaction buffer (Southern Cross Biotechnology, Cape Town, South Africa). The only exception was the 16S rRNA PCR, which utilized 5 ng μl^−1^ of template DNA, and the reaction mixture excluded bovine serum albumin. Amplicons were purified using 20 U µl^−1^ Exonuclease I (LTC Tech South Africa Proprietary Limited) and 1 U µl^−1^ alkaline phosphatase (LTC Tech South Africa Proprietary Limited, Johannesburg, South Africa) (Werle et al. 1994).

The purified PCR products were sequenced using the original forward and reverse primers, as well as the ABI BigDye® Terminator v3.1 Cycle Sequencing Kit (Thermo Fisher Scientific, Waltham MA, United States) and ABI Prism 3130xl sequencer (Thermo Fisher Scientific). Electropherograms were analyzed using Chromas version 2.4.1 (Technelysium, Queensland, Australia) and consensus sequences created using BioEdit version 7.2.5 (Hall 1999). The sequences for all PCR products were deposited into the NCBI database under accession numbers OQ458741-OQ458755 for 16S rRNA, with links to BioProject accession numbers PRJNA937154 for *atpD*, PRJNA937057 for *recA*, PRJNA938292 for *dnaK*, PRJNA938296 for *glnII* and PRJNA938285 for *rpoB*.

Multiple sequences alignments for the various gene regions were compiled. For this purpose, a 16S rRNA dataset was constructed using the EZTaxon server (http://www.ezbiocloud.net/eztaxon accessed on 10th September 2023) (Kim et al. 2012) and BLASTn searches against the nucleotide collection (nr/nt) database of the National Centre for Biotechnology Information (NCBI; http://www.ncbi.nlm.nih.gov) (Johnson et al. 2008). The 16S rRNA dataset was then aligned using the online webserver of MAFFT version 7 (Katoh et al. 2005) with the Q-INS-I strategy to account for RNA secondary structures (Katoh and Toh 2008). For the five housekeeping genes, we constructed individual datasets containing sequences generated in the current study, as well as those for the type strains of known species as listed on the List of Prokaryotes with Standing in Nomenclature (LPSN, available at https://lpsn.dsmz.de/, accessed on 15th September 2023) that captures all *Mesorhizobium* type strains irrespective of their names’ valid publication (Euzéby 1997). We also included representatives of the unnamed species most closely related to our strains that are included in the Genome Taxonomy Database (https://gtdb.ecogenomic.org/ accessed on 1st April 2024) (Parks et al. 2022). Codon-based alignments were then manually constructed in BioEdit for the housekeeping gene datasets.

The aligned sequences were subjected to maximum-likelihood phylogenetic analysis in RAxML version 8.0.20 using the General Time Reversible (Tavaré, 1986) substitution model with gamma correction for among site rate heterogeneity, and a proportion of invariable sites. Branch support was estimated using the same model parameters and bootstrapping with 1000 pseudo-replications. For concatenation of the five housekeeping datasets (i.e. to allow multilocus sequence analysis; MLSA), we used FASconCAT-G version 1.04 (Kück and Meusemann 2010). Phylogenetic analysis of the latter dataset was conducted as before, with individual model parameters set for each gene partition. All phylogenetic trees were visualized using Mega version 11.0.13 and edited with Inkscape version 0.92.3 (Inkscape Project, 2020. *Inkscape*, Available at: https://inkscape.org).

### Whole genome sequence analysis

Five isolates were selected for genome sequence analysis. These included three isolates (Cs1299R1N1, Cs1299R1N3, Cs1321R2N1) recovered from *C. sericea* grown in Brakputs rhizosphere soils, one isolate from *C. sericea* grown in Kamieskroon rhizosphere soil (i.e. Cs1330R2N1^Ts^) and one isolate from *L. diffusa* grown in Brakputs rhizosphere soil (i.e. Ld1326N3^Ts^). From these isolates, high-quality DNA was isolated using the Promega Wizard Genomic DNA purification kit (Madison, USA). Whole genome sequencing was done using Illumina MiSeq at the Biotechnology Platform of the Agricultural Research Council, Onderstepoort, Pretoria, South Africa.

Raw sequences were subjected to FastQC version 0.11.7 (Andrews 2010) and Trim Galore version 0.6.6 (https://github.com/FelixKrueger/TrimGalore) for adaptor removal and quality filtering. The trimmed sequences were then assembled using SPAdes software version 3.13.0 (Bankevich et al. 2012). BBtools software version 38.90 (https://jgi.doe.gov/data-and-tools/software-tools/bbtools/) was used to calculate the genome sequencing coverage (Bushnell 2014). Barrnap software version 0.9 (https://github.com/tseemann/barrnap) was used to predict the location of the 16S rRNA, 5S rRNA and 23S rRNA genes (Seemann 2013). TRNAscan-SE version 2.0 was used to predict transfer RNA (tRNA) genes (Chan et al. 2021; Chan and Lowe 2019). The proposed genomes representing novel nomenclatural types (see below) were taxonomically assigned using the GTDB-Tk version 2.4.0 that assigns taxonomic positions relative to the database (GTDB-Tk reference) in KBase (Chaumeil et al. 2020; Arkin et al. 2018). Our new genome sequences, together with those for relevant type strains and/or close relatives obtained from the NCBI and the GTDB; https://gtdb.ecogenomic.org/, databases were used for average nucleotide identity (ANI) analysis using the BLAST-based method (ANIb) implemented in the webserver of JSpecies version 4.1.1 (http://jspecies.ribohost.com/jspeciesws/#analyse) (Richter et al. 2016).

We further employed the up-to-date bacterial core gene (UBCG version 3.0) pipeline (Na et al. 2018) that predicts, extracts and aligns 92 core genes using Prodigal version 2.6.3, Hmmsearch version 3.1b2 and MAFFT version 7.320 as dependencies (Na et al. 2018). The 92 genes were concatenated and subjected to ML analyses using IQ-TREE release 2.2.2.6 (Nguyen et al. 2015). Branch support was estimated by performing Ultrafast Bootstrap approximation (UFBoot) of 1000 pseudoreplicates (Hoang et al. 2018). The Shimodaira–Hasegawa approximate likelihood ratio test (SH-aLRT) was also performed for 1000 replicates per branch (Guindon et al. 2010). The Gene Support Index (GSI, a metric indicating the number of individual gene trees supporting each a particular branch), calculated by the UBCG pipeline was also recorded as an additional indication of genealogical concordance among individual genes (Venter et al. 2017; Na et al. 2018). The taxon selection for the UBCG analysis included genomes classified as *Mesorhizobium *sensu stricto according to van Lill et al. (2024).

### Phenotypic characterization

All isolates examined in this study were subjected to various phenotypic tests. These included growth evaluation of the isolates on YMA at 15 ºC, 28 ºC, 35 ºC and 45 ºC, respectively, for 7 days. We also determined growth at 28 ºC for 7 days in yeast mannitol broth (YMB) at adjusted pH 4–12, as well as on YMA supplemented with 0.5, 1.0 and 1.5% (w/v) of NaCl, respectively.

Additional tests were conducted on the five isolates used for genome analysis (i.e. Cs1299R1N1, Cs1299R1N3, Cs1321R2N1, Cs1330R2N1^Ts^ and Ld1326^Ts^). These included motility tests which involved stab-inoculation of transparent test tubes containing semi-solid (0.4%) YMA (Howieson and Dilworth 2016). The five isolates were also subjected to carbon source utilization tests using Biolog GN2 (bioMereux, France) and substrate assimilation tests using API 20NE (bioMereux, France). For evaluating heavy metal resistance of our five isolates, 100 µl of fresh YMB cultures were spread onto YMA plates. Then, sterile filter paper discs (6 mm in diameter, Whatman International Limited, Maidstone England) were aseptically placed on to the YMA plates and 10 µl of sterile metal solution were immediately impregnated on to the filter paper discs and incubated at 28 ^o^ C for 7 days. The metal concentrations were 1.5 mM MnCl_2_∙4H_2_O, 0.07 mM NiSO_4_∙6H_2_O, 0.05 mM Pb(C_2_H_3_O_2_)_2_∙3H_2_O, 0.07 Mm CoSO_4_∙7H_2_O, 0.7 mM ZnSO_4_∙7H_2_O and 0.08 mM CuSO_4_∙5H_2_O (Tomova et al. 2015; Alzoreky and Nakahara 2003; Berrada et al. 2012).

### Nodulation tests

The nodulation capabilities of all the rhizobia examined in this study were evaluated on both *C. sericea* and *L. diffusa*. Seeds for both legume species were germinated as described above, after which two seedlings were planted in 1.9 L sized plastic pots containing sterile sand. For inoculation, YMB cultures were incubated for 5 days at 28 °C, after which volumes of 2 ml were taken and applied directly onto the two plants in the respective pots. Plants were maintained (28° C for 14 h daytime and 15° C for 10 h nighttime) and watered three times a week using nitrogen free Hoagland solution (Howieson and Dilworth 2016). Control samples (where seedlings were not inoculated) for each legume species were included. Plants were grown for 6–8 weeks after which nodules were harvested.

Once harvested, nodules were surface sterilized as described above. Each nodule was squashed in 300 µl sterile water and the resulting suspensions were streaked onto YMA and incubated at 28 °C. Bacterial cultures were then re-streaked onto fresh YMA and isolates originating from single colonies were subjected DNA extraction, and *rpoB* PCR sequencing as described above. These sequences were then compared against those of the original strains to ascertain if the nodules were induced by the respective strains.

### Phylogenetic analysis of symbiotic genes

For all isolates included in this study, a portion of *nifH* was PCR amplified with primers PolF and PolR as described previously (Poly et al. 2001). The amplicons were purified and sequenced as above, and raw sequences submitted to NCBI as BioProject accession number PRJNA940066. These sequences were then included in a dataset containing the *nifH* sequences extracted from the genome sequences determined in the current study. The dataset also included the *nifH* gene sequences from published genomes of other strains by making use of the GTDB; https://gtdb.ecogenomic.org/, and the kb_gtdbtk version 1.0.0 application in Kbase (https://narrative.kbase.us/) (Arkin et al. 2018). Additionally, the dataset included nearly all the *nifH* sequences identified with BLASTn in NCBI’s nucleotide database.

Sequences for *nodC* and *nodA* were also extracted from the genomes determined in the current study. Datasets containing these sequences were then developed in the same way as for the *nifH* gene. Moreover, relevant sequences for *Bradyrhizobium japonicum* USDA6^T^ and or *Bradyrhizobium canariense* BTA-1^ T^ / CCBAU 51257 were used for outgroup purposes. For all the three symbiotic loci, a codon-based aligment was manually produced in BioEdit version 7.2.5. These datasets were reduced by representing identical sequences as haplotypes (Supplementary Tables S5, S6 and S7) using DnaSP version 6. 12.03 (Rozas et al. 2017). Thereafter, the aligned dataset was subjected to RAxML analysis as described above.

## Results

### Soil properties and rhizobial isolates

The four rhizosphere soil samples (Ld1326, Cs1299, Cs1321 and Cs1330) from *L. diffusa* and *C. sericea*, respectively, were subjected to various soil properties analyses (Table 2). They were classified as sandy or loamy sand with pH ranging from 4.7 (extremely acidic) for the Cs1330 Kamieskroon sample to 7.1 (neutral) for the Cs1299 Brakputs sample. The lowest value for nitrates were also detected in the Cs1330 soil sample (0.1 mg kg^−1^ NO_3_^−^), while sample Cs1321 from Brakputs contained most nitrates (i.e. 1.5 mg kg^−1^ NO_3_^−^). A total of 15 rhizobial isolates were recovered from the two legumes species grown using their respective soil samples. Three isolates were obtained from *L. diffusa* nodules (Ld1326N3^Ts^, Ld1326N4, Ld1326N5) grown in soil sample Ld1326 (Brakputs), while 12 came from the nodules of *C. sericea* (Cs1299R1N1, Cs1299R1N2, Cs1299R1N3, Cs1299R2N1, Cs1299R2N2, Cs1299R2N3, Cs1299R2N4, Cs1321R1N1, Cs1321R2N1, Cs1321R3N1 and Cs1321R3N2) grown in samples Cs1299, Cs1321 (Brakputs) and Cs1330R2N1^Ts^ from soil sample Cs1330 (Kamieskroon).

**Table 2:** Rhizobial isolates originating from the *Lessertia diffusa* and *Calobota sericea* rhizosphere soils examined, as well as their characteristics

Soil properties	Composite soil sample			
	Ld1326 (Brakputs)	Cs1299 (Brakputs)	Cs1321 (Brakputs)	Cs1330 (Kamieskroon)
Rhizophere legume	*L. diffusa*	*C. sericea*	*C. sericea*	*C. sericea*
Soil type	Loamy sand	Sandy	Sandy	Loamy sand
Soil pH	6.2	7.1	6.3	4.7
pH ratings*	Slightly acidic	Neutral	Slightly acidic	Extremely acidic
NO_3_^−^ (mg kg^−1^)	0.52	0.85	1.50	0.10
NH_4_^+^ (mg kg^−1^)	7.40	7.42	6.88	6.79
Total carbon (%)	0.42	0.22	0.26	0.27
Total nitrogen (%)	0.04	0.02	0.02	0.04
Number of rhizobial isolates (isolate numbers)	3 (Ld1326N3^Ts^, Ld1326N4 and Ld1326N5)	7 (Cs1299R1N1, Cs1299R1N2, Cs1299R1N3, Cs1299R2N1, Cs1299R2N2, Cs1299R2N3 and Cs1299R2N4)	4 (Cs1321R1N1, Cs1321R2N1, Cs1321R3N1 and Cs1321R3N2)	1 (Cs1330R2N1^Ts^)

^*^pH ratings are described according to (Bruce and Rayment 1982)

### Delineation of presumptive *Mesorhizobium* species

**Fig. 1:**
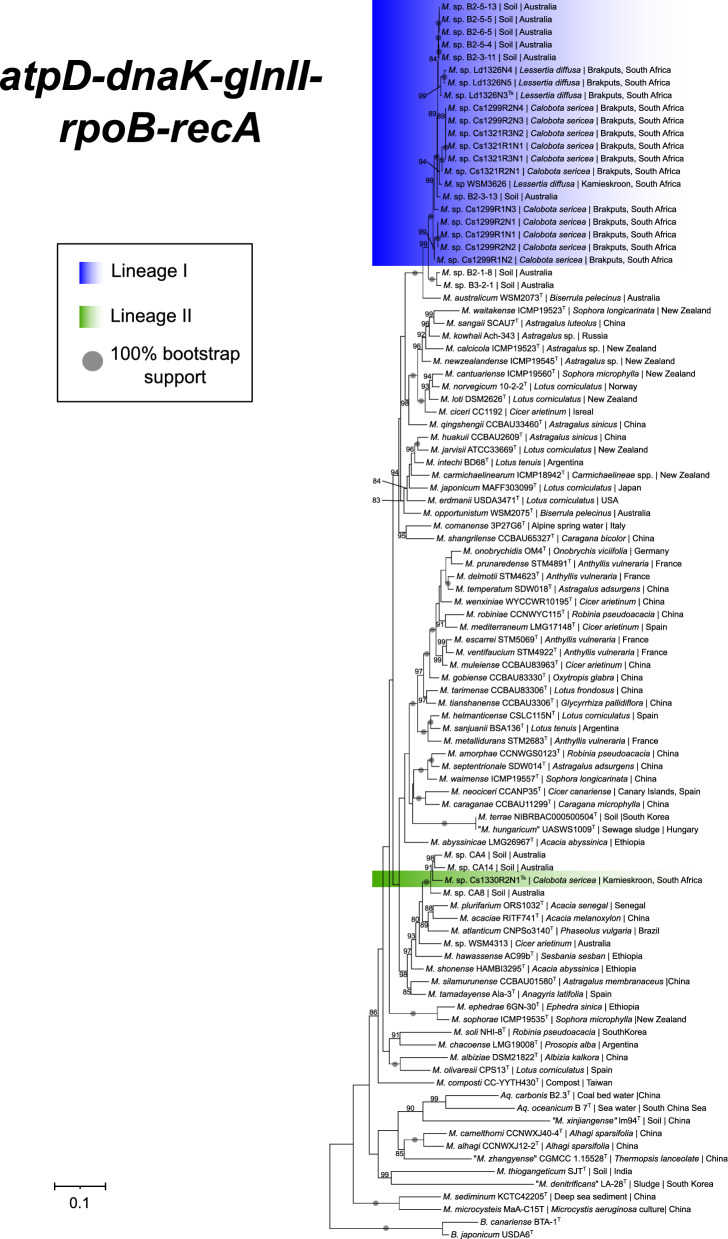
Maximum-likelihood phylogeny inferred from the concatenated *atpD*, *glnII*, *dnaK*, *recA* and *rpoB* housekeeping gene sequences. Strains highlighted in blue and green colors represent Lineages I and II respectively inferred from the strains identified in this study and their grouping on individual housekeeping gene phylogenies. Sequences of *Bradyrhizobium japonicum* USDA6^T^ and *Bradyrhizobium canariense* BTA-1^T^ were used for outgroup purposes. Type strains or sequences are indicated as T or Ts and those strains whose names are not validly published according to LPSN are indicated with inverted commas. Bootstrap support values were inferred from 1000 pseudoreplicates and only values greater than 80% are indicated at the nodes, bifurcations with bootstrap support of 100% are indicated as circles on the particular branch. The scale bar indicates nucleotide substitutions per site. GenBank accession numbers are listed in (Supplementary Table S1)

Comparison of the 16S rRNA gene sequences obtained for the 15 isolates examined in this study with other available sequences included in the NCBI database revealed that they were members of *Mesorhizobium* as defined in the GTDB’s current release (Parks et al. 2022). Phylogenetic analysis of the 16S rRNA alignment (consisting of 1010 nucleotides and 101 taxa) separated our isolates into two lineages (Lineages I and II; Supplementary Fig. S1), albeit with low bootstrap support. Because of this low resolution, we constructed five housekeeping gene datasets containing our sequences together with those of the type strains of *Mesorhizobium*. The *dnaK* and *atpD* dataset consisted, respectively, of 249 and 360 nucleotides with 90 and 93 taxa. The *glnII*, *recA* and *rpoB* datasets, respectively, contained 261, 291 and 414 for 87, 98 and 97 taxa. In all the datasets, two relevant sequences from *Bradyrhizobium* were used for outgroup purposes. Information about accession numbers of all the references and outgroup taxa sequences used is provided in Supplementary Table S1. Phylogenetic analysis of all 5 of these datasets, as well as the concatenated five-gene dataset also separated our 15 isolates into the same lineages as recovered from the 16S rRNA phylogeny (Fig. 1; Supplementary Fig. S2).

Lineage I included 14 of our isolates (Ld1326N3^Ts^, Ld1326N4, Ld1326N5, Cs1299R1N1, Cs1299R1N2, Cs1299R1N3, Cs1299R2N1, Cs1299R2N2, Cs1299R2N3, Cs1299R2N4, Cs1321R1N1, Cs1321R2N1, Cs1321R3N1 and Cs1321R3N2). Based on the five-gene concatenated phylogeny and the *atpD*, *dnaK* and *glnII* gene trees, Lineage 1 also included strain WSM3626 previously obtained from the nodules of *Lessertia diffusa* in Kamieskroon (Gerding et al. 2012, Gerding et al. 2013), as well as strains (B2-6-5, B2-3-13, B2-3-11, B2-5-5, B2-5-4 and B2-5-13) with whole genome sequences in the GTDB (Fig. 1; Supplementary Fig. S2). The GTDB strains were all recovered from soil samples in Badgingarra area in Western Australia (NCBI BioProject PRJNA549135). Overall, Lineage I corresponded to GTDB unnamed species “*Mesorhizobium* sp000513935”, and was most closely related to the GTDB strains B3-2-1 and B2-1-8, also isolated from soil in Badgingarra (NCBI BioProject PRJNA549135). The clade containing Lineage I and these two strains had a sister group relationship with *Mesorhizobium australicum* WSM2073^T^. However, the *recA* and *rpoB* phylogenies clustered Lineage I with *M. australicum* WSM2073^T^ and strains B3-2-1 and B2-1-8 (Supplementary Fig. S2).

Lineage II was singularly represented by isolate Cs1330R2N1^Ts^ that was positioned as a separate taxon compared to the other *Mesorhizobium* strains in all of the phylogenies examined (Supplementary Fig. S2). For example, in the *rpoB* and *recA* trees, Cs1330R2N1^Ts^ clustered with strain CA4 (GTDB unnamed species “*Mesorhizobium* sp020164955”) in a clade including strains CA8 (GTDB unnamed species “*Mesorhizobium* sp020164955”) and CA14 (GTDB unnamed species “*Mesorhizobium* sp020165195”), while the *dnaK* tree clustered it with strain CA8 in a larger clade containing strains CA14, CA4, WSM 4313 (GTDB unnamed species “*Mesorhizobium* sp002294725”) and *Mesorhizobium atlanticum* CNPSo3140^T^. Strains CA4, CA8 and CA14 originated from soil samples from the Canna region in Australia (NCBI BioProject PRJNA549135), while strain WSM 4313 originated from *Cicer arietinum* near the Moree region in Australia (NCBI BioProject PRJNA399611). Such a varied clustering pattern was thus indicative that isolate Cs1330R2N1^Ts^ represent a novel species, which according to the five-gene phylogeny is most closely related to unnamed species GTDB species CA4 and CA14 with a bootstrap of 91% supporting this branch (Fig. 1). Based on the results of these phylogenetic analyses, we accordingly regarded Lineages I and II as putative species hypotheses, which we ultimately name *Mesorhizobium argentiipisi* sp. nov. Cs1330R2N1^Ts^ and *Mesorhizobium salmacidum* sp. nov. Ld1326N3^Ts^, respectively (see below).

### Whole genome support for the species hypotheses

Evaluation of additional evidence for the two species hypotheses delineated above involved genome sequence analyses of five representative isolates (Table 3). These included the Lineage II isolate Cs1330R2N1^Ts^, and four isolates spanning the diversity in Lineage I (i.e. Ld1326N3^Ts^, Cs1299R1N1, Cs1299R1N3, and Cs1321R2N1). The respective genome assemblies consisted of 6.4 to 6.9 million bases (Mb), made up of 98–366 contigs, with 62.3–62.9% G + C content. The sequencing coverage ranged between 117 and 225x, with N50 values of between 34,099 bases (bp) and 409,983 bp. The number of tRNA genes ranged between 53 and 59, with a complete 16S rRNA gene detected in all assemblies. The genome data thus conformed to the minimum quality requirements for use in taxonomic studies (Chun et al. 2018; De Lajudie et al. 2019; Hedlund et al. 2022) and were deposited at the NCBI database with accession numbers GCA_037179605.1 (Ld1326N3^Ts^), GCA_037179655.1 (Cs1299R1N1), GCA_037179625.1 (Cs1299R1N3), GCA_037179725.1 (Cs1321R2N1) and GCA_037179585.1 (Cs1330R2N1^Ts^). Additionally, in accordance with the requirements for the SeqCode (Hedlund et al 2022), we submitted the corresponding raw sequence data to NCBI’s Sequence Read Archive (SRA) under the BioProject accession number PRJNA1028499. The two genomes designated as the nomenclatural types were registered under the SeqCode Registry lists (https://registry.seqco.de/registers/r:eyqg8ked and https://registry.seqco.de/registers/r:n_eruou3).

**Table 3:** Descriptive features for the five draft *Mesorhizobium* genomes assembled in this study

Genome statistics	*M. salmacidum*				*M. argentiipisi*
	Ld1326N3^Ts^	Cs1299R1NI	Cs1299R1N3	Cs1321R2N1	Cs1330R2N1^Ts^
Size (bp)	6,432,310	6,990,524	6,502,566	6,415,389	6,951,081
Number of contigs	123	161	98	366	150
Largest contig	523,142	722,678	1,133,841	130,770	611,319
N50	211,414	409,983	340,975	34,099	188,352
Coverage	225x	128x	151x	153x	117x
% G + C	62.6	62.3	62.6	62.5	62.9
% Completeness	99.51	99.51	99.51	98.69	99.1
% Contamination	0.29	1.43	1.02	0.27	0.0
% 16S rRNA gene completeness	100	100	100	100	100
Conspecific to GTDB representative	GCF_000513935.1	n/a	n/a	n/a	n/a
Number of standard amino acids decoded by tRNA elements	20	20	20	20	20
Genbank accessions	GCA_037179605.1	GCA_037179655.1	GCA_037179625.1	GCA_037179725.1	GCA_037179585.1
SRA accession	SRR26412432	SRR26412431	SRR26412430	SRR26412429	SRR26412428
SeqCode registry no	https://registry.seqco.de/registers/r:n_eruou3	–	–	–	https://registry.seqco.de/registers/r:eyqg8ked

The two species hypotheses identified above were also supported using the UBCG pipeline (Supplementary Fig. S3). From the 92 genes tree, our Lineage I strains (Ld1326N3^Ts^, Cs1299R1N1, Cs1299R1N3, and Cs1321R2N1) grouped with strain WSM3626 representing “*Mesorhizobium* sp000513935”. These data also confirmed that GTDB unnamed species “*Mesorhizobium* sp006439515” (strains B3-2-1 and B2-1-8) is the closest relative of Lineage I. Similarly, the Lineage II strain Cs1330R2N1^Ts^ formed part of a larger clade containing strains CA14, C4 and C8.

**Fig. 2:**
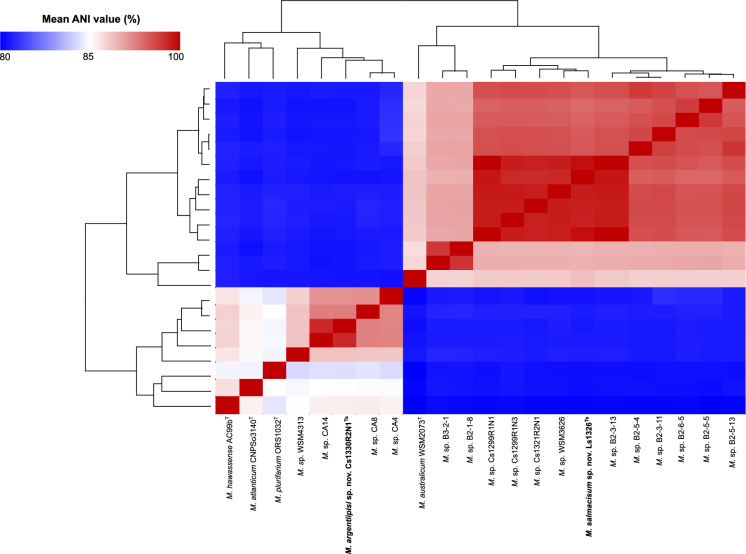
A heatmap showing the Average Nucleotide Identity (ANI) similarity between the representative strains from the delineated groups in this study and the closest relatives, alongside a dendogram generated from the similarity matrix. ANIb pairwise values were calculated using the BLAST algorithm in JSpecies, ANIb, and higher values indicate higher average similarity between two genomes. For the full result see Supplementary Table S3. Type strains or sequences are indicated as (T or Ts)

In terms of genome sequence similarity, strain Ld1326N3^Ts^ shared > 95% ANI identity with all other members of Lineage I, i.e. unnamed GTDB species “*Mesorhizobium* sp000513935” (Fig. 2; Supplementary Tables S2 and S3). Analysis using JSpecies showed that the four genomes sequenced in the current study (i.e. Ld1326N3^Ts^, Cs1299R1N1, Cs1299R1N3, and Cs1321R2N1) and strains WSM3626, B3-2-13, B2-5-5, B2-5-13, B2-6-5, B2-5-4 and B2-3-11) shared ANIb values ranging from 95.12 to 97.33%. However, when comparing Lineage I with its close relatives much lower ANIb values were recorded, i.e. 82.31–91.02% for *M. australicum* WSM2073^T^, 82.76–92.47% for GTDB species *Mesorhizobium* sp. B3-2-1 and 82.73–92.55% for GTDB species *Mesorhizobium* sp. B2-1-8. These identity patterns are thus congruent with those proposed for the members of the same species (i.e. and ANI value of approximately > 95%) (Arahal, 2014).

In the case of Lineage II isolate Cs1330R2N1^Ts^, analysis with GTDB-Tk confirmed its uniqueness, with closest GTDB genome being that of strain CA14 or “*Mesorhizobium* sp020165195” (Supplementary Table S2). As expected for members of different species (Arahal 2014), JSpecies-based ANIb comparisons of Cs1330R2N1^Ts^ yielded values below 95% (i.e. 93.98% for strain CA8, 93.95% for strain CA4; and 93.92% for strain CA14) (Fig. 2; Supplementary Table S3).

### Phenotypic characteristics

Most of isolates from Lineage I displayed growth at pH 6–10, although variations were observed (e.g. Cs1299R2N3 and Cs1299R2N4 could grow at pH 4) (Supplementary Table S4). Lineage II isolate Cs1330R2N1^Ts^ could grow at pH 4–10. In terms of temperature, all isolates grew optimally at 28 ºC (Supplementary Table S4). The strains of Lineage I grew at 15–35 ºC, except isolates Cs1299R1N3, Cs1299R2N3 and Cs1299R2N2 which also showed growth at 37 ºC. Lineage II isolate Cs1330R2N1^Ts^ could grow at 15–37 ºC. Additionally, all isolates grew optimally in the presence of 0.5% NaCl, with reduced growth at 1% and 1.5% NaCl (Supplementary Table S4).

The isolates representing Lineage I generally displayed the same results for carbon source utilization tests with Biolog and substrate assimilation tests with API 20NE (Supplementary Table S4). Overall, these isolates appeared to be metabolically versatile and capable assimilating or utilizing most of the compounds tested, a trend that was also observed for Lineage II isolate Cs1330R2N1^Ts^. Interestingly, the Lineage I isolates could utilize stachyose and D-raffinose while Lineage II isolate Cs1330R2N1^T^ could not. Furthermore, all the five strains were resistant to the metals tested (Supplementary Table S4).

### Nodulation tests

All the fifteen strains considered in this study were capable of nodulating their specific hosts plants (*L. diffusa* and *C. sericea*) by the eighth week after inoculation in a controlled environment. The harvested nodules were pink in colour upon dissecting, which is indicative of leghaemoglobin formation and effective nitrogen fixation (Murphy-Bokern et al. 2017). Additionally, the *Mesorhizobium* strains isolated from *C. sericea* nodules could not induce nodulation of *L. diffusa*. One of the isolates from *L. diffusa*, Ld1326N3^T^, induced the formation of a small number of white nodules with white interiors on *C. sericea*. The latter indicates that the nodules were ineffective in nitrogen fixation, which was also confirmed by stunted yellow plants. In all cases, the pure cultures originating from the nodules observed had *rpoB* sequences similar to those that were initially used as inoculum.

### Phylogenetic analysis of symbiotic genes

The symbiotic gene datasets constructed contained all publicly available *nifH*, *nodC* and *nodA* sequences for *Mesorhizobium*. Also, the *nifH* dataset included relevant sequences obtained for 13 of the isolates included in this study (accession number PRJNA940066). In the case of *nodC* and *nodA*, the datasets included sequences for only the five isolates subjected to genome sequencing. All of the latter contained single copies of the *nodA* and *nodC*, except for the genome of isolate Ld1326N3^Ts^ (originating from *L. diffusa* nodules in Brakputs) that harboured three copies of *nodA*. We subsequently also detected three copies of *nodA* in the genome of WSM3626 previously obtained from *L. diffusa* nodules in Kamieskroon (Gerding et al. 2012). Nevertheless, the *nifH* alignment consisted of 748 taxa grouped into 106 haplotypes and 150 nucleotides, the *nodA* alignment contained 584 taxa (120 haplotypes) and 393 nucleotides, and the *nodC* alignment contained 459 taxa (99 haplotypes) and 345 nucleotides.

Unlike the housekeeping gene phylogenies examined, trees inferred from the *nifH* and *nodC* sequences did not group strains according to their species identities (Figs. S4, S5; Supplementary Tables S5-S6). Instead, these genes grouped our strains based on their geographic origins. For example, the *nifH* phylogeny (Fig. S4) separated the isolates into two sister groups, with one containing 11 isolates (all from *C. sericea* nodules obtained using soils from Brakputs) and the other containing isolates obtained from *L. diffusa* nodules using Brakputs soil and from *C. sericea* nodules obtained using soils from Kamieskroon. The latter group also included strain WSM3626 and our isolate, Ld1326N3^Ts^, that were represented by the same *nifH* haplotype. However, the entire assemblage of 14 South African isolates formed a distinct and separate cluster in the *nifH* tree. In other words, the *nifH* tree grouped the South African members of Lineages I and II together and separate from other Lineage I members originating from soil sampled in Australia https://gtdb.ecogenomic.org/, and from strain WSM4313 originating from *Cicer arietinum* in Australia https://www.ncbi.nlm.nih.gov/datasets/genome/. As expected (Muema et al. 2022), all of our *nifH* sequences also grouped closely with those recovered from a previous metabarcoding study of rhizosphere soils of *C. sericea*, *L. diffusa* and several other legumes in Succulent Karoo region particularly Brakputs and Kamieskroon (Supplementary Fig. S7).

In the *nodA* phylogeny (Fig. S6; Supplementary Table S7), our isolates were separated into three broad groups that were all made up of strains exclusively isolated from South Africa. The largest of these groups included root-nodule bacteria obtained from *L. diffusa*, *L. incana*, *L. excisa*, *L. herbacea*, *L. pauciflora*, *L. annularis*, *L. microphylla* and *L. capitate* sampled in the Fynbos, Succulent Karoo or Nama Karoo biomes in the Western Cape, Northern Cape or Eastern Cape Provinces in South Africa (Gerding et al. 2012). One of the three *nodA* sequences of Ld1326N3^Ts^ (haplotype 35) and WSM3626 (haplotype 45) also clustered within this large group. The second and third *nodA* sequences of these two strains were found within the two remaining groups, with Ld1326N3^Ts^ (haplotype 37) and WSM3626 (haplotype 47) clustering with *M. neociceri* CCANP35^T^ isolated from *Cicer canariense* in Spain (León-Barrios et al. 2021), albeit with no bootstrap support. Similar to the unique grouping observed in the *nifH* and *nodC* phylogenies of the South African strains separate from other Lineage I isolates (i.e. B2-6-5 and WSM4313), we also see this distinction between the strains in the *nodA* phylogeny.

## Discussion

Here we used a stepwise polyphasic approach based on the analyses of genealogical concordance of multiple protein coding genes involved in housekeeping functions, as well as genome data and phenotypic characters to robustly delineate Lineages I and II, both of which represent new species of *Mesorhizobium* (Venter et al. 2017). Firstly, clusters of isolates representing the two lineages were consistently supported by phylogenies inferred from the *atpD*, *glnII*, *dnaK, rpoB* and *recA* genes. Additional lines of evidence for them were then sought by exploring genomic and phenotypic data. Although this grouping pattern was mostly supported by 16S rRNA, this gene generally lacked resolving power at species level (Ramasamy et al. 2014; Rajendhran and Gunasekaran 2011; Fox et al. 1992). Nevertheless, for representatives of the two putative species we observed genome-based similarity patterns congruent with the expectations for strains of the same or different species (Goris et al. 2007; Konstantinidis and Tiedje 2005). In other words, all of the bacteria included in Lineage I shared ANI values exceeding 95%, while all comparisons between the Lineage I and II and other known *Mesorhizobium* species yielded values below 95%. For these two taxa, we accordingly propose the names *Mesorhizobium salmacidum* sp. nov. and *Mesorhizobium argentiipisi* sp. nov. respectively (for protologue descriptions, see Tables 4 and 5).

**Table 4:** Protologue description for *Mesorhizobium salmacidum* sp. nov

Genus name	*Mesorhizobium*
Species name	*Mesorhizobium salmacidum*
Specific epithet	*salmacidum*
Species status	sp. nov
Species etymology	sal.ma.ci'dum, L. neut. adj. *salmacidum*, salty or brackish, referring to the brackish water of the region of isolation
Description of a new taxon and diagnostic traits	They are gram negative, rod-shaped, and motile bacteria. The colony morphology is white to creamy colour, often with a circular or irregular form that is elevated. Able to tolerate a pH range between 6 and 9. Grows well at < 0.5% NaCl concentration but can also have reduced growth at both 1% and 1.5% NaCl concentration. Can grow at temperature ranges between 15º to 35º with an optimum growth at 28 ºC. The strain tested negative for the activity of nitrate reduction to nitrite, arginine dehydrogenase, β-glucosidase, tryptophan deaminase but positive for urease and β-galactosidase. Utilizes L-arabinose, Potassium gluconate, trisodium citrate, D-maltose, D-trehalose, D-cellubiose, D-gentiobiose, sucrose, D-turanose, α-D-lactose, D-melibiose, β-methyl-D glucoside, D-salicin, N-acetyl-D glucosamine, N-acetyl-β-D mannosamine, N-acetyl-D galactosamine, α-D-glucose, D-mannose, D-fructose, D-galactose, D-fucose, L-fucose, L-rhamnose, D-sorbitol, D-mannitol, D-arabitol, inositol, glycerol, D-glucose6-PO4, D-fructose6-PO4, D-aspartic acid, gelatin, Glycyl-L-proline, L-alanine, L-arginine, L-glutamic acid, L-histidine, L-pyroglutamic acid, pectin, D-galacturonic acid, L-galactonic acid, lactone, D-gluconic acid, D-glucuronic acid, glucuronamide, D-lactic acid methyl ester, L-lactic acid, citric acid, α-keto-glutaric acid, D-malic acid, L-malic acid, bromo-succinic acid, tween 40, γ-Amino-butyric acid, β-Hydroxy-D, L-Butyric acid, acetoacetic acid, acetic acid, formic acid as sole source of carbon, but not L-arabinose, capric acid, adipic acid, stachyose, D-raffinose, N-acetyl neuraminic acid, inosine, D-serine, L-serine, propionic acid, α-Keto-butyric acid, P-Hydroxyphenyl acetic acid among others. It is resistant to ampicillin, gentamicin, kanamycin, chloramphenicol, tetracycline spectinomycin, neomycin, penicillin and streptomycin, but sensitive to erythromycin. Resistant to Ni, Zn, Pb, Co, Mn, and Cu
Country of origin	South Africa
Region of origin	Northern Cape Province, Brakputs, Succulent Karoo biome
Source of isolation	Root nodule of* Lessertia diffusa*
Genome accession number	GCA_037179605.1^Ts^
Genome status	Draft
Genome size	6411 Kbp
N50	211,414
GC mol%	62.63
Number of strains in this lineage	14
Source of isolation of non-type genome	Legume root nodules
Designation of type genome	Ld1326N3^Ts^
SeqCode registry list	https://registry.seqco.de/registers/r:n_eruou3

**Table 5:** Protologue description for *Mesorhizobium argentiipisi* sp. nov

Genus name	*Mesorhizobium*
Species name	*Mesorhizobium argentiipisi*
Specific epithet	*argentiipisi*
Species status	sp. nov
Species etymology	ar.gen.ti.i.pi'si (L. neut. n. *argentum*, silver; N.L. gen. n. *pisi*, of a pea; N.L. gen. n. *argentiipisi*, of the silver pea, referring to the common name, Silver pea, of *Calobota sericea*
Description of a new taxon and diagnostic traits	It is gram a negative, rod-shaped, and motile bacteria. The colony morphology is white to creamy colour, often with a circular or irregular form that is elevated. Able to tolerate a pH range between 4 and 10. Grows well at < 0.5% NaCl concentration but can also have reduced growth at both 1% and 1.5% NaCl concentration. Can grow at temperature ranges between 15º to 37º with an optimum growth at 28 ºC. The strain tested positive for the activity of nitrate reduction to nitrite, arginine dehydrogenase, urease, β-galactosidase, and β-glucosidase but not tryptophan deaminase. Utilizes Potassium gluconate, trisodium citrate, dextrin, D-maltose, D-trehalose, D-cellubiose, D-gentiobiose, sucrose, D-turanose, α-D-lactose, D-melibiose, β-methyl-D glucoside, D-salicin, N-acetyl-D glucosamine, N-acetyl-β-D mannosamine, N-acetyl-D galactosamine, α-D-glucose, D-mannose, D-fructose, D-galactose, 3-methyl glucose, D-fucose, L-fucose, L-rhamnose, D-sorbitol, D-mannitol, D-arabitol, inositol, glycerol, D-glucose6-PO4, D-fructose6-PO4, D-aspartic acid, gelatin, Glycyl-L-proline, L-alanine, L-arginine, L-aspartic acid, L-glutamic acid, L-histidine, L-pyroglutamic acid, pectin, D-galacturonic acid, L-galactonic acid, lactone, D-gluconic acid, D-glucuronic acid, glucuronamide, mucic acid, quinic acid, D-saccharic acid,, methyl pyruvate, D-lactic acid methyl ester, L-lactic acid, citric acid, α-keto-glutaric acid, D-malic acid, L-malic acid, bromo-succinic acid, tween 40, γ-Amino-butyric acid, α-hydroxy-butyric acid, β-Hydroxy-D, L-Butyric acid, acetoacetic acid, propionic acid, acetic acid, formic acid as sole source of carbon, but not L-arabinose, capric acid, adipic acid, stachyose, D-raffinose, N-acetyl neuraminic acid, inosine, D-serine, L-serine or P-Hydroxyphenyl acetic acid. Sensitive to erythromycin, ampicillin, gentamicin, kanamycin, chloramphenicol, tetracycline spectinomycin, neomycin, penicillin and streptomycin. Resistant to Ni, Zn, Pb, Co, Mn, and Cu
Country of origin	South Africa
Region of origin	Northern Cape Province, Kamieskroon, Succulent Karoo biome
Source of isolation	Root nodule of *Calobta cericea*
Genome accession number	GCA_037179585.1^Ts^
Genome status	Draft
Genome size	6951 Kbp
N50	188,352
GC mol%	62.9
Number of strains in this lineage	1
Designation of type strain	Cs1330R2N1^Ts^
SeqCode registry list	https://registry.seqco.de/registers/r:eyqg8ked

Various laboratory assays showed that the two new *Mesorhizobium* species are metabolically versatile and capable of growth under a diversity of environmental conditions. Both appear to be tolerant to heavy metals (Ni, Zn, Pb, Co, Mn and Cu), a trait previously shown to be linked to soil pH, biogeography as well as the species identity of strains (Brígido et al. 2017). Although limited data are generally available for *Mesorhizobium* species (Brígido et al. 2017, Velez et al. 2017, Wani and Khan 2013, Hu Frisk et al. 2022), most species particularly, the chickpea microsymbionts seem to be tolerant to Zn, Pb and others (Brígido et al. 2017). The fact that our two new species were capable of growth across a wide temperature range was also in line with reports for other *Mesorhizobium* species with optimal growth typically at temperatures between 25 ºC and 30 ºC (De Meyer et al. 2015; Sannazzaro et al. 2018; Jung et al. 2020). Additionally, they could also thrive in media across a wide pH range (pH 6–10, and pH 4 in the case of *Mesorhizobium argentiipisi* sp. nov.), which is similar to reports for closely related species such as *M*. *atlanticum* CNPSo3140^T^, *M*. *hawassense* AC99b^T^ that are capable of growth in more acidic conditions (pH 4–4.5) and *M. australicum* WSM2073^T^ that can grow at pH 5.5 to 9.0 (Nandasena et al. 2009; Degefu et al. 2013; Helene et al. 2019). The two new species also seem capable of thriving at salinities ranging from 0.5% NaCl to a maximum of 1.5%, which is higher than reports for closely related taxa such as *M. hawassense* AC99b^T^ capable of growth in the presence of 0.5% NaCl (Degefu et al. 2013), and *Mesorhizobium atlanticum* CNPSo3140^T^ and *Mesorhizobium plulifarium* ORS1032^T^ that are able to grow in medium containing 1.0% NaCl (de Lajudie et al. 1998; Helene et al. 2019).

Both of *M. salmacidum* sp. nov. and *M argentiipisi* sp. nov. are related to bacteria previously isolated in soils or legumes from Western Australia. Species most closely related to *M*. *argentiipisi* sp. nov. are represented by strains isolated from soils in the Canna region (NCBI BioProject PRJNA549135), while those most closely related to *M. salmacidum* sp. nov. are represented by strains from soils in Badgingarra (NCBI BioProject PRJNA549135), as well as *M. australicum*, which nodulates *Biserrula pelecinus* cultivated in Northam (Nandasena et al. 2009, 2007). This pasture legume was introduced in the nineties from the Mediterranean region into Australia where native rhizobia rapidly gained the ability to effectively nodulate it by acquiring the requisite symbiotic genes from an inoculant strain used to commercially cultivate it (Nandasena et al. 2006, 2009). In addition to isolates from *C. sericea* and *L. diffusa* in Brakputs and Kamieskroon, *M. salmacidum* sp. nov. also included six isolates originating from soil in Badgingarra in Western Australia (NCBI BioProject PRJNA549135). The latter likely reflects the similar climatic and environmental conditions prevailing in the two regions, with both mainly receiving winter rainfall and acidic, arid and nutrient poor soils (Gerding et al. 2013).

The nodulation abilities of the *M. salmacidum* sp. nov. and *M*. *argentiipisi* sp. nov. isolates examined in this study did not appear to be associated with their species identities, but rather the identity of their host legumes. Isolates obtained from *L. diffusa* nodules could effectively nodulate only this legume species and not *C. cericea*, despite representing members of the same species. The same was also true for the *C. cericea* isolates. These two legumes species are unrelated with *L. diffusa* belonging to the tribe Galageae and *C. sericea* belonging to the tribe Crotalarieae legume species (Balkwill and Balkwill 1999) (https://powo.science.kew.org/taxon/urn:lsid:ipni.org:names:21907-1). This apparent pattern host-specificity is likely driven by factors such as legume flavonoids (Walker et al. 2020), and host-specificity also has been reported for *Mesorhizobium* species from other legumes including *Cicer*, *Lotus*, *Biserrula*, *Anthyllis* and *Caragana* (León-Barrios et al. 2021; Chen et al. 2008; Greenlon et al. 2019).

Phylogenetic analysis of the *nifH*, *nodA* and *nodC* genes clustered all of the *Mesorhizobium* strains examined in this study together and separate from strains from other parts of the world. This could be attributed to variations in the symbiotic islands, given their mobile nature of the genetic elements and sensitivity to flavonoids (Geddes et al. 2020; Ling et al. 2016). For instance, three copies of *nodA* locus were observed in *L. diffusa* symbionts compared to *C. sericea*. Such variations could explain the nodulation tests which proved the inability of *C. sericea* symbionts to nodulate *L. diffusa* species, while *L. diffusa* symbionts formed two small white nodules on *C. sericea*, a further confirmation of incompatibility of their symbionts. This means that although the *C. sericea* strains having different core genomes and species identities, their symbiotic genes are conserved (Fall et al. 2021; Dludlu et al. 2018). Similar to *C. sericea*, the *Mesorhizobium* microsymbionts of legume species *Astragulas sinicus* carried similar *nodA* locus but their chromosomes were different (Zhang et al. 2000).

In recent years, it was impossible to publish new species from South Africa due to regulation constraints that govern sharing of biological resources internationally (Alexander et al. 2021; Hamer et al. 2021), which is a requirement of the International code of nomenclature of prokaryotes (ICNP) (Parker et al. 2019). This study therefore has demonstrated the benefit of the recently developed SeqCode to name and publish new species by utilizing their presentative genome sequences as nomenclatural types. This approach was recently used to delineate and publish the novel *Salinibacter* species *Sal. pepae*, *Sal. pampae* sp. nov. and *Sal. abyssi* sp. nov (Viver et al. 2023), and several *Mesorhizobium* species associated with *Vachellia karroo*, an acacia legume species native to Southern Africa (van Lill et al. 2024).

## Conclusions

This study demonstrated the use of SeqCode, using genome sequences as nomenclatural types (Whitman et al. 2022) to describe novel rhizobia species associated with legumes. Furthermore, understanding rhizobia associated with *C. sericea* and *L. diffusa* in the SKB in South Africa is vital because it explains the wide distribution of these legumes, which are invaluable sources of animal fodder in the SKB and other surrounding biomes (Samuels et al. 2016; Müller et al. 2017, 2021). Such knowledge can inform the management to develop conservation strategies of *C. sericea* and *L. diffusa* shrubs in the era of changes in climate with more persisted severe droughts and invasive plant species. Moreover, the *Mesorhizobium* species identified in this study showed tolerance to different heavy metals tested. This means that these species have the potential for bioremediation of heavy metals in the environment associated with mining activities in this region. Simultaneously, these species have the potential for rehabilitation of the mining ores, hence can play a significant role in conserving the environment.

## Supplementary Information

Below is the link to the electronic supplementary material.

Supplementary Fig. S1 Maximum likelihood phylogeny based on 16S rRNA gene sequences data for the fifteen isolates investigated reference and outgroup strains sequences. Strains indicated in blue and green colors represent those investigated in this study. The DNA sequences for *Bradyrhizobium japonicum* USDA6^T^ and *Bradyrhizobium canariense* BTA-1^T^ were used for outgroup purposes, while the rest of the *Mesorhizobium* strains sequences were used as reference sequences. The accession numbers for the sequences are indicated in brackets. Type strains or sequences are indicated as T or Ts. Bootstrap values were inferred using 1000 pseudo replicates with only those values >50% indicated at the nodes. The scale bar indicates the number of nucleotide changes per site. (PDF 70 KB)

Supplementary Fig. S2 Maximum-likelihood phylogenies of *atpD*, *glnII*, *dnaK*, *recA* and *rpoB* house-keeping genes for the fifteen isolates being investigated, reference and outgroup strains sequences. Strains highlighted in blue and green colours represent Lineages I and II respectively representing those investigated in this study. Sequences of *Bradyrhizobium japonicum* USDA6^T^ and *Bradyrhizobium canariense* BTA-1^T^ were used for outgroup purposes. Type strains or sequences are indicated as T or Ts and those strains whose names are not validly published according to LPSN are indicated with inverted commas. Bootstrap support values were inferred from 1000 pseudoreplicates and only values greater than 50% are indicated at the nodes. The scale bar indicates nucleotide substitutions per site. GenBank accession numbers are listed in (Supplementary Table S1). (PDF 3622 KB)

Supplementary Fig. S3 UBCG phylogenomic tree of all our five isolates and representative strains of described and undescribed *Mesorhizobium* sensu stricto in the GTDB with genome accession numbers indicated in brackets. Strains highlighted in blue and green colours represent Lineages I and II respectively representing those investigated in this study with the proposed type sequences of novel species indicated in bold. Type strains or sequences are indicated as T or Ts and those strains whose names are not validly published according to LPSN are indicated with inverted commas. Bootstrap support values were inferred from 1000 pseudoreplicates and only values greater than 80% are indicated at the nodes, bifurcations with bootstrap support of 100% are indicated as circles on the particular branch. Gene support index (GSI) values ‘n’ (highlighted in red in the tree) demonstrate how many of the bacterial core genes support that bifurcation. Only values lower than the maximum value are demonstrated next to bootstrap values (i.e., < 92). USDA 6*T* was used as the outgroup. The scale bar indicates nucleotide substitutions per site. (PDF 158 KB)

Supplementary Fig. S4 Maximum-likelihood phylogenetic tree constructed using *nifH* gene sequences of the strains investigated in this study, reference, and outgroup strains sequences. Strains highlighted in blue and green colours represent those investigated in this study. Sequences of *Bradyrhizobium japonicum* USDA6^T^ was used for outgroup purposes. Type strains or sequences are indicated as (T or Ts). Bootstrap support values were inferred from 1000 pseudoreplicates and only values greater than 50% are indicated at the nodes. The scale bar indicate nucleotide substitutions per site. The accession numbers for the sequences are indicated in Supplementary Table S5. (PDF 87 KB)

Supplementary Fig. S5 Maximum-likelihood phylogenetic tree constructed using *nodC* gene sequences of the strains investigated in this study, reference, and outgroup strains sequences. Strains indicated in blue and purple colours represent those investigated in this study. Sequences of *Bradyrhizobium japonicum* USDA6T and *Bradyrhizobium canariense* CCBAU 51257 were used for outgroup purposes. Type strains or sequences are indicated as T or Ts. Bootstrap support values were inferred from 1000 pseudo replicates and only values greater than 50% are indicated at the nodes. The scale bar indicates nucleotide substitutions per site. The accession numbers for the sequences are indicated in Supplementary Table S6. (PDF 104 KB)

Supplementary Fig. S6 Maximum-likelihood phylogenetic tree constructed using *nodA* gene sequences of the strains investigated in this study, reference, and outgroup strains sequences. Strains highlighted in blue and green colours represent those investigated in this study. Sequences of *Bradyrhizobium japonicum* USDA6^T^ and *Bradyrhizobium canariense* BTA-1^T^ were used for outgroup purposes. Type strains sequences are indicated as T or Ts. Bootstrap support values were inferred from 1000 pseudo replicates and only values greater than 50% are indicated at the nodes. The scale bar indicates nucleotide substitutions per site. The accession numbers for the sequences are indicated in Supplementary Table S7. (PDF 103 KB)

Supplementary Fig. S7 Maximum-likelihood phylogenetic tree constructed using *nifH* gene sequences of the strains investigated in this study, *nifH* metabarcoding sequences of operational taxonomic units (OTUs), reference strains, and outgroup strains sequences. Strains indicated in blue and purple colours represent those investigated in this study. Sequences of *Bradyrhizobium japonicum* USDA6^T^ and *Bradyrhizobium diazoefficiens* USDA110^T^ were used for outgroup purposes. The isolates marked in bold are sequences of OTUs from the legume rhizosphere soils, with legume species hosts indicated in square brackets, followed the by locations of rhizosphere soils and seeds sampling. The accession numbers for the sequences are indicated in brackets. Type strains are indicated as T or Ts. Bootstrap support values were inferred from 1000 pseudo replicates and only values greater than 50% are indicated at the nodes. The scalebars indicates nucleotide substitutions per site. (PDF 113 KB)

Supplementary file8 (XLSX 83 KB)

## Data Availability

All data generated or analysed from this study are included in this published article. For example, the whole genome sequences of the five representative isolates used in this study have been deposited in the NCBI database with accession numbers: GCA_037179605.1 (Ld1326N3^Ts^), GCA_037179655.1 (Cs1299R1N1), GCA_037179625.1 (Cs1299R1N3), GCA_037179725.1 (Cs1321R2N1), and GCA_037179585.1 (Cs1330R2N1^Ts^). Their corresponding raw sequence data were also submitted to NCBI’s Sequence Read Archive (SRA) under the BioProject accession number PRJNA1028499. The sequences for all PCR products were deposited into the NCBI database under accession numbers OQ458741-OQ458755 for 16S rRNA, with links to BioProject accession numbers PRJNA937154 for atpD, PRJNA937057 for recA, PRJNA938292 for dnaK, PRJNA938296 for glnII, PRJNA938285 for rpoB, and PRJNA940066 for nifH. Any additional data are provided as supplementary information files.
