# Supplementary figures and images for: Mesorhizobium salmacidum sp. nov. and Mesorhizobium argentiipisi sp. nov. are symbionts of the dry-land forage legumes Lessertia diffusa and Calobota sericea
Source: Antonie Van Leeuwenhoek. 2025 Feb 12;118(3):54. doi: 10.1007/s10482-025-02063-2 (PMC11814006; doi:10.1007/s10482-025-02063-2)

16S rRNA

- Lineage I
- Lineage II

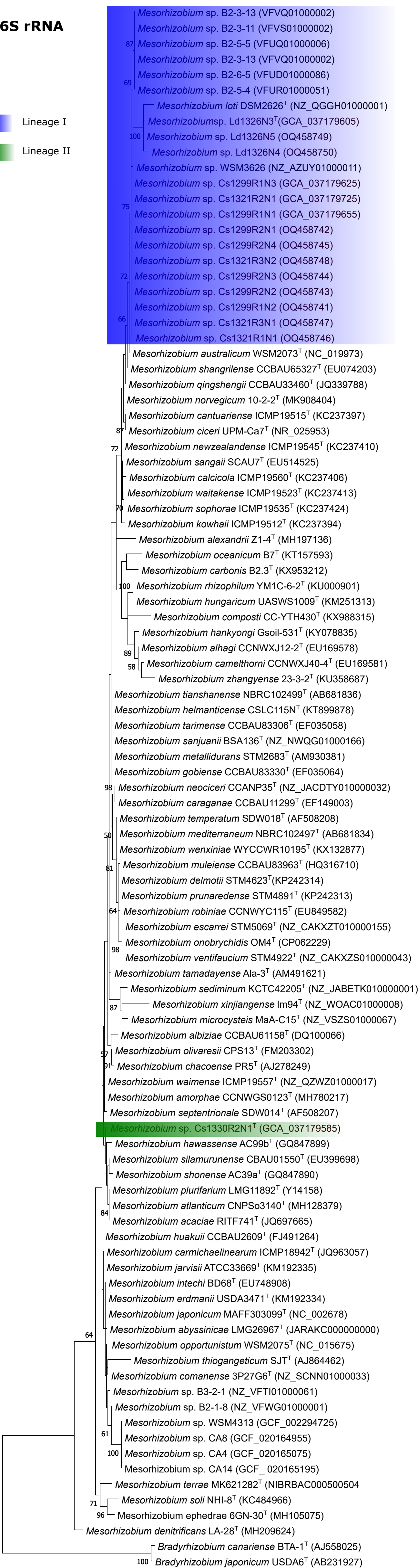

0.10

Supplement: Supplementary file 1 — Supplementary Fig. S1 Maximum likelihood phylogeny based on 16S rRNA gene sequences data for the fifteen isolates investigated reference and outgroup strains sequences. Strains indicated in blue and green colors represent those investigated in this study. The DNA sequences for Bradyrhizobium japonicum USDA6T and Bradyrhizobium canariense BTA-1T were used for outgroup purposes, while the rest of the Mesorhizobium strains sequences were used as reference sequences. The accession numbers for the sequences are indicated in brackets. Type strains or sequences are indicated as T or Ts. Bootstrap values were inferred using 1000 pseudo replicates with only those values >50% indicated at the nodes. The scale bar indicates the number of nucleotide changes per site. (PDF 70 KB) [file 10482_2025_2063_MOESM1_ESM.pdf]

atpD

dnaK

glnII

rpoB

recA

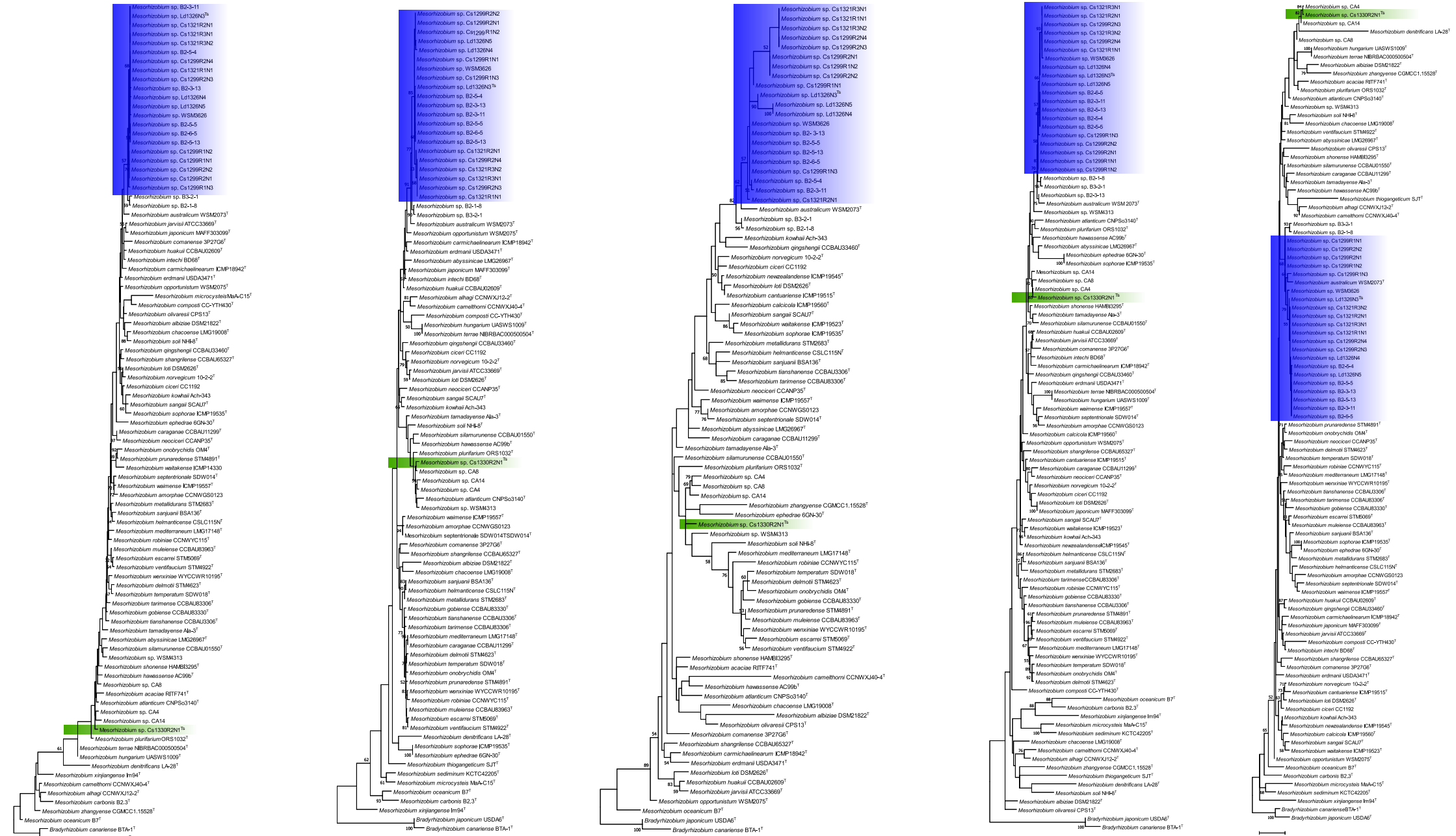

Lineage I Lineage II

Supplement: Supplementary file 2 — Supplementary Fig. S2 Maximum-likelihood phylogenies of atpD, glnII, dnaK, recA and rpoB house-keeping genes for the fifteen isolates being investigated, reference and outgroup strains sequences. Strains highlighted in blue and green colours represent Lineages I and II respectively representing those investigated in this study. Sequences of Bradyrhizobium japonicum USDA6T and Bradyrhizobium canariense BTA-1T were used for outgroup purposes. Type strains or sequences are indicated as T or Ts and those strains whose names are not validly published according to LPSN are indicated with inverted commas. Bootstrap support values were inferred from 1000 pseudoreplicates and only values greater than 50% are indicated at the nodes. The scale bar indicates nucleotide substitutions per site. GenBank accession numbers are listed in (Supplementary Table S1). (PDF 3622 KB) [file 10482_2025_2063_MOESM2_ESM.pdf]

# UBCG

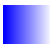

Lineage I

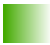

Lineage II

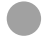

100% bootstrap support

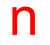

Gene support index (GSI)  
value <92

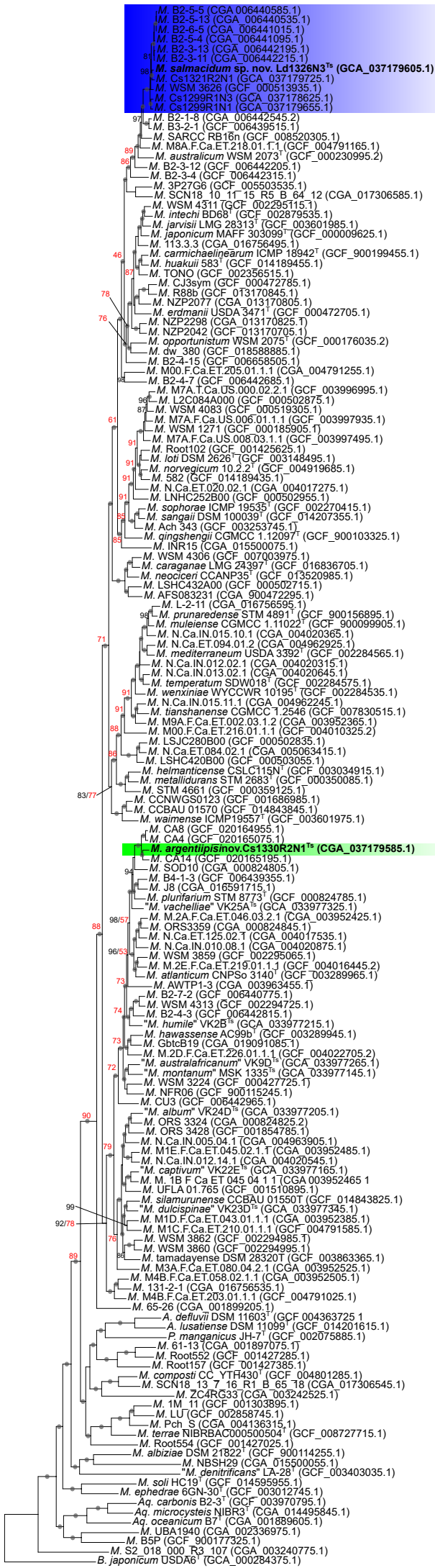

Supplement: Supplementary file 3 — Supplementary Fig. S3 UBCG phylogenomic tree of all our five isolates and representative strains of described and undescribed Mesorhizobium sensu stricto in the GTDB with genome accession numbers indicated in brackets. Strains highlighted in blue and green colours represent Lineages I and II respectively representing those investigated in this study with the proposed type sequences of novel species indicated in bold. Type strains or sequences are indicated as T or Ts and those strains whose names are not validly published according to LPSN are indicated with inverted commas. Bootstrap support values were inferred from 1000 pseudoreplicates and only values greater than 80% are indicated at the nodes, bifurcations with bootstrap support of 100% are indicated as circles on the particular branch. Gene support index (GSI) values ‘n’ (highlighted in red in the tree) demonstrate how many of the bacterial core genes support that bifurcation. Only values lower than the maximum value are demonstrated next to bootstrap values (i.e., < 92). USDA 6T was used as the outgroup. The scale bar indicates nucleotide substitutions per site. (PDF 158 KB) [file 10482_2025_2063_MOESM3_ESM.pdf]

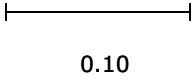

Supplement: Supplementary file 4 — Supplementary Fig. S4 Maximum-likelihood phylogenetic tree constructed using nifH gene sequences of the strains investigated in this study, reference, and outgroup strains sequences. Strains highlighted in blue and green colours represent those investigated in this study. Sequences of Bradyrhizobium japonicum USDA6T was used for outgroup purposes. Type strains or sequences are indicated as (T or Ts). Bootstrap support values were inferred from 1000 pseudoreplicates and only values greater than 50% are indicated at the nodes. The scale bar indicate nucleotide substitutions per site. The accession numbers for the sequences are indicated in Supplementary Table S5. (PDF 87 KB) [file 10482_2025_2063_MOESM4_ESM.pdf]

nodC

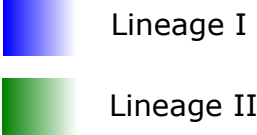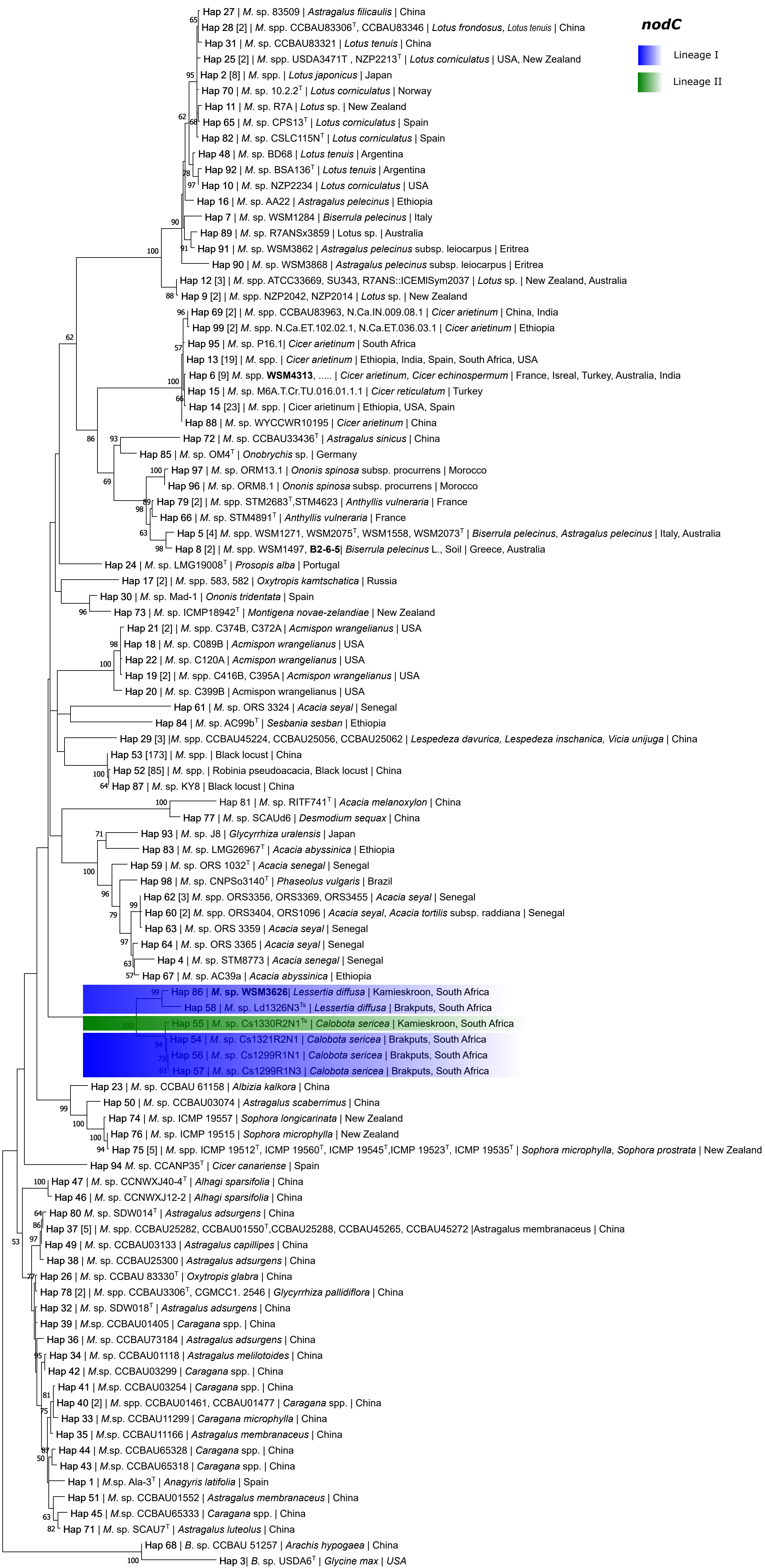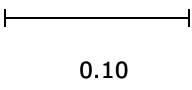

Supplement: Supplementary file 5 — Supplementary Fig. S5 Maximum-likelihood phylogenetic tree constructed using nodC gene sequences of the strains investigated in this study, reference, and outgroup strains sequences. Strains indicated in blue and purple colours represent those investigated in this study. Sequences of Bradyrhizobium japonicum USDA6T and Bradyrhizobium canariense CCBAU 51257 were used for outgroup purposes. Type strains or sequences are indicated as T or Ts. Bootstrap support values were inferred from 1000 pseudo replicates and only values greater than 50% are indicated at the nodes. The scale bar indicates nucleotide substitutions per site. The accession numbers for the sequences are indicated in Supplementary Table S6. (PDF 104 KB) [file 10482_2025_2063_MOESM5_ESM.pdf]

nodA

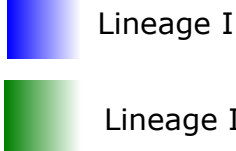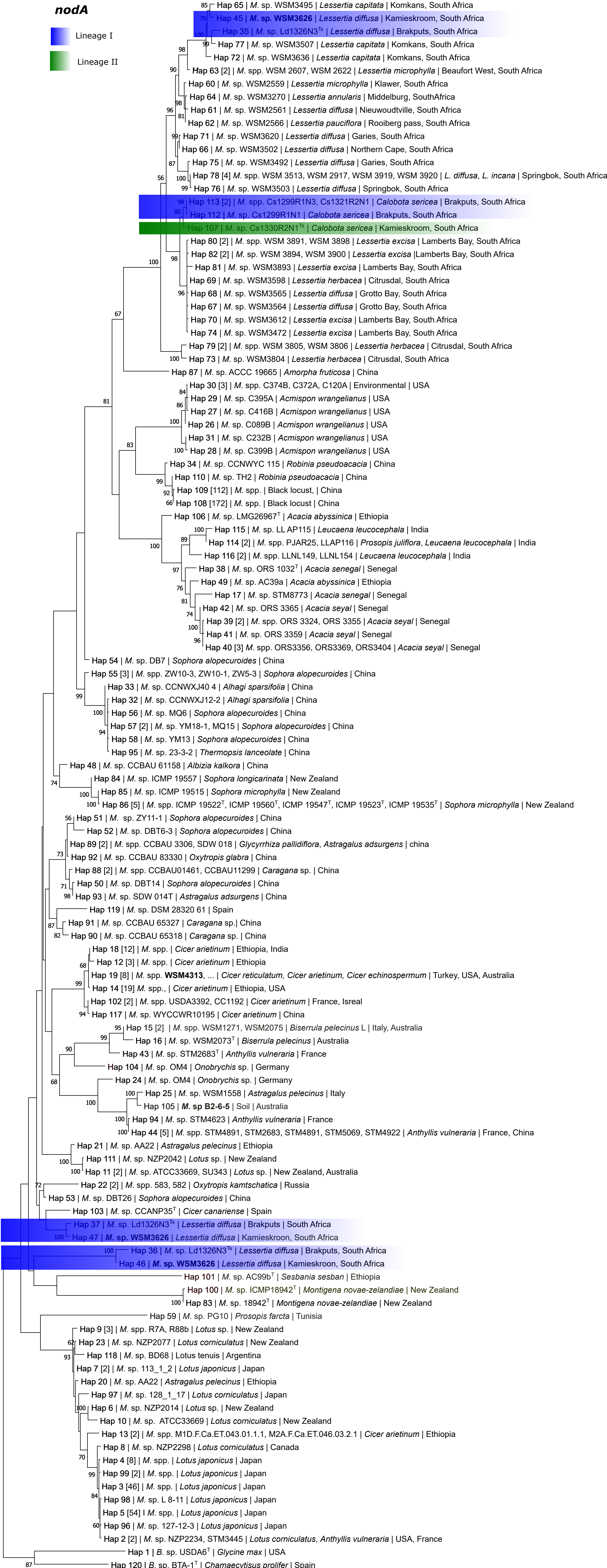

0.20

Supplement: Supplementary file 6 — Supplementary Fig. S6 Maximum-likelihood phylogenetic tree constructed using nodA gene sequences of the strains investigated in this study, reference, and outgroup strains sequences. Strains highlighted in blue and green colours represent those investigated in this study. Sequences of Bradyrhizobium japonicum USDA6T and Bradyrhizobium canariense BTA-1T were used for outgroup purposes. Type strains sequences are indicated as T or Ts. Bootstrap support values were inferred from 1000 pseudo replicates and only values greater than 50% are indicated at the nodes. The scale bar indicates nucleotide substitutions per site. The accession numbers for the sequences are indicated in Supplementary Table S7. (PDF 103 KB) [file 10482_2025_2063_MOESM6_ESM.pdf]

nifH (nodules + soils)

Lineage I

Lineage II

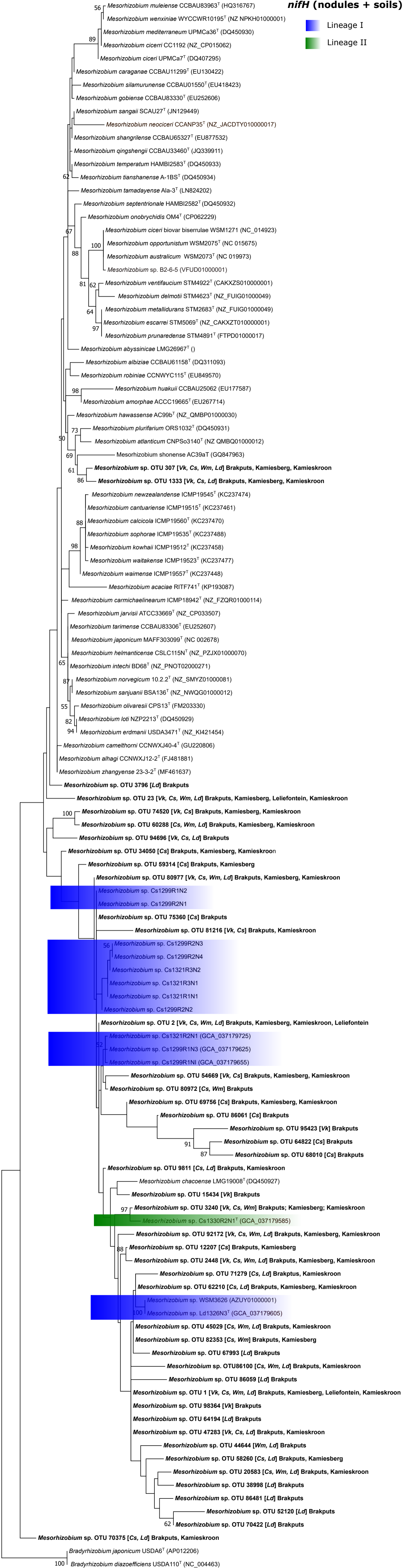

0.02

Supplement: Supplementary file 7 — Supplementary Fig. S7 Maximum-likelihood phylogenetic tree constructed using nifH gene sequences of the strains investigated in this study, nifH metabarcoding sequences of operational taxonomic units (OTUs), reference strains, and outgroup strains sequences. Strains indicated in blue and purple colours represent those investigated in this study. Sequences of Bradyrhizobium japonicum USDA6T and Bradyrhizobium diazoefficiens USDA110T were used for outgroup purposes. The isolates marked in bold are sequences of OTUs from the legume rhizosphere soils, with legume species hosts indicated in square brackets, followed the by locations of rhizosphere soils and seeds sampling. The accession numbers for the sequences are indicated in brackets. Type strains are indicated as T or Ts. Bootstrap support values were inferred from 1000 pseudo replicates and only values greater than 50% are indicated at the nodes. The scalebars indicates nucleotide substitutions per site. (PDF 113 KB) [file 10482_2025_2063_MOESM7_ESM.pdf]
